# Finding an alternative diagnosis does not justify increased use of CT-pulmonary angiography

**DOI:** 10.1186/1471-2466-13-9

**Published:** 2013-02-07

**Authors:** Subani Chandra, Pralay K Sarkar, Divay Chandra, Nicole E Ginsberg, Rubin I Cohen

**Affiliations:** 1 Division of Pulmonary, Critical Care and Sleep Medicine, The Long Island Jewish Medical Center, The Hofstra North Shore-LIJ School of Medicine, New Hyde Park, New York, NY, 11040, USA; 2 Division of Pulmonary, Sleep and Critical Care Medicine, University of Pittsburgh Medical Center, Pittsburgh, PA, 15213, USA

## Abstract

**Background:**

The increased use of computed tomography pulmonary angiography (CTPA) is often justified by finding alternative diagnoses explaining patients’ symptoms. However, this has not been rigorously examined.

**Methods:**

We retrospectively reviewed CTPA done at our center over an eleven year period (2000 – 2010) in patients with suspected pulmonary embolus (PE). We then reviewed in detail the medical records of a representative sample of patients in three index years – 2000, 2005 and 2008. We determined whether CTPA revealed pulmonary pathology other than PE that was not readily identifiable from the patient’s history, physical examination and prior chest X-ray. We also assessed whether the use of pre-test probability guided diagnostic strategy for PE.

**Results:**

A total of 12,640 CTPA were performed at our center from year 2000 to 2010. The number of CTPA performed increased from 84 in 2000 to 2287 in 2010, a 27 fold increase. Only 7.6 percent of all CTPA and 3.2 percent of avoidable CTPAs (low or intermediate pre-test probability and negative D-dimer) revealed previously unknown findings of any clinical significance. When we compared 2008 to 2000 and 2005, more CTPAs were performed in younger patients (mean age (years) for 2000: 67, 2005: 63, and 2008: 60, (p=0.004, one–way ANOVA)). Patients were less acutely ill with fewer risk factors for PE. Assessment of pre-test probability of PE and D-dimer measurement were rarely used to select appropriate patients for CTPA (pre-test probability of PE documented in chart (% total) in year 2000: 4.1%, 2005: 1.6%, 2008: 3.1%).

**Conclusions:**

Our data do not support the argument that increased CTPA use is justified by finding an alternative pulmonary pathology that could explain patients’ symptoms. CTPA is being increasingly used as the first and only test for suspected PE.

## Background

Computed tomography pulmonary angiography (CTPA) is the preferred method to confirm or exclude a PE. However the non-selective use of CTPA has several disadvantages. These include long term risks of exposure to high doses of radiation and a small but definite risk of kidney injury due to intravenous contrast. Moreover, CTPA is an expensive test and often leaves behind a trail of incidental findings of indeterminate implication that further increase health care costs [1,2]. Even with CTPA’s technological advances, easy availability and ever shorter scan times, the assessment of clinical pre-test probability remains central to the diagnosis of PE and ideally should influence the initial choice of diagnostic testing [3-5]. However, it remains unclear how frequently pre-test probability algorithms are followed in daily clinical practice [6-8]. Furthermore, while the number of CTPA has increased dramatically, the majority of CTPA do not show the presence of PE. Nevertheless, the increase in the use of CTPA is often justified by the discovery of hitherto unknown pathological etiologies [9-13]; however, review of the literature would indicate that this has not been rigorously examined. In this study we examined the patterns of CTPA use over an eleven year period at a single center and assessed whether the use of CTPA was justified in those with low or intermediate pre-test probability by the finding of an alternative diagnosis that might have explained the patient’s symptoms.

## Methods

We determined the total number of CTPA performed in patients >18 years in age, and their results (as either positive or negative for PE) for each year from 2000–2010 in a 500 bed, academic teaching hospital in New York City. The study was approved by the North Shore-Long Island Jewish Health System’s institutional review board which waived the need for informed consent.

We studied in detail, the medical records of patients who underwent CTPA in three index years 2000, 2005, and 2008. We chose the year 2000 as a baseline reference year, the year 2005 was the first calendar year after the hospital obtained an additional CT scanner, and the year 2008 followed the publication of Prospective Investigation of Pulmonary Embolism Diagnosis II (PIOPED II) and its recommendations [3-5,14]. We reviewed all available records for the year 2000 (74 records) and a random sample representative of all CTPA performed in 2005 and 2008. A total of 850 studies were reviewed for 2005 and 2008. We excluded CT chest angiograms performed for other reasons such as aortic dissection. Demographic data, clinical presentation, risk factors for thromboembolic disease, documentation of pre-test probability of PE, use of therapeutic anticoagulation, and results of CTPA were abstracted from patients’ medical records. Since increased use of CTPA may be replacing that of V/Q scans, we also determined the number of V/Q scans performed for the diagnosis of PE from 2000 to 2010.

### Assessment of pre-test probability of PE

We assigned pre-test probability to all subjects using the Revised Geneva Score (RGS) [15]. At the time of assignment of pre-test probability, the investigators were blinded to the results of the CTPA. Based on the RGS we assigned subjects to low (RGS 0–3), intermediate (RGS 4–10), and high (RGS ≥ 11) pre-test probability categories. CTPA performed on patients who were in the low or intermediate pre-test probability category and had a negative D-dimer were considered avoidable.

### D-dimer measurement

In 2000 and 2005, D-dimer was measured by quantitative latex agglutination and in 2008 by ELISA (Enzyme-linked immunosorbent assay) with fluorescence (ELFA) (VIDAS ® D-dimer Exclusion TM, bioMérieux, Marcy l'Etoile, France). The D-dimer assay is readily available in our hospital and the results are reported within 30 minutes.

### CT pulmonary angiography

In 2000, CTPAs were performed on single or 4 – slice scanners (HiSpeed, GE Healthcare, UK). In 2005 another 4-slice CT scanner (HiSpeed, GE Healthcare, UK) was installed and the existing CT scanners were upgraded to 16-slice CT machines (LightSpeed, GE Healthcare, UK). In 2008, all CTPA were being performed on 16-slice scanners (LightSpeed and BrightSpeed, GE Healthcare, UK). Central PE was defined as PE in the pulmonary trunk, right or left main pulmonary arteries or lobar arteries while PE in segmental or sub-segmental branches were considered peripheral.

### Sample size calculation

We used an estimate of the proportion of patients who had a CTPA and were in the low probability group for the sample size calculations. Statistically, the most conservative calculation occurs if 50% of patients fall into this category. We imposed a constraint to estimating within 5 percentage points of the expected proportion since the sample size for the years 2005 and 2008 was so large. For a sample size of 385, a two-sided 95% confidence interval for a single proportion is no wider than ± 5.0% from an expected overall proportion of 50% (i.e. 45% to 55%). Based on this estimate a random sample of 383 and 393 CTPA were reviewed for 2005 and 2008 respectively. We reviewed all available CTPA results (74 of 84) for the year 2000.

### Statistical analyses

All analyses were performed using Stata 11.1 (StataCorp LP, College Station, TX). Chi-square tests were used for categorical data, while one-way ANOVA was used for continuous variables. A two-tailed *p*-value < 0.05 was considered statistically significant.

## Results

**Figure 1:**
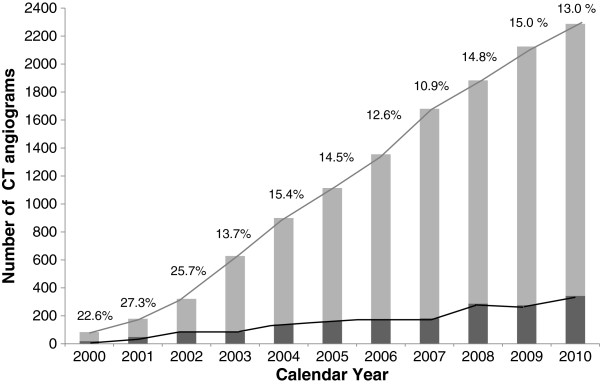
**The number of CTPA performed per calendar year from 2000 to 2011.** The dark bars and percentages are the CTPA that were positive for PE.

**Figure 2:**
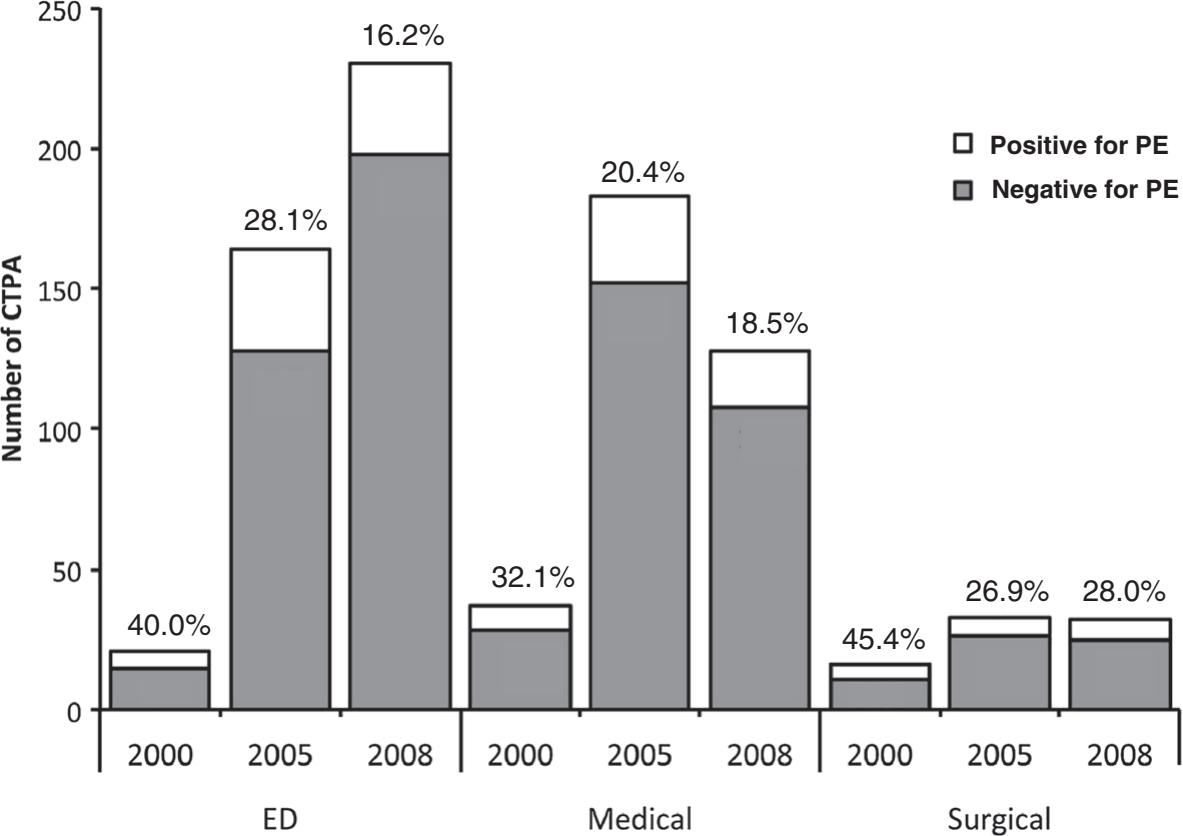
**Number of CTPA by hospital department for the index years 2000, 2005 and 2008.** The largest increase occurred in the emergency department (ED) which also had the steepest decline in positive yield (% above bars).

Over the study period (2000 to 2010), there was a 27-fold increase in the total number of CTPA (Figure 1) without a corresponding increase in yield. The total number of CTPA in the three index years 2000, 2005 and 2008 were 84, 1114 and 2287 respectively. While the number of CTPA ordered increased in all departments, the ED had the largest increase. The numbers of scans performed in the ED per 100 visits were 0.14 in 2000, 1.82 in 2005 and 2.58 in 2008. The ED also had the steepest decline in the percentage of scans positive for PE (Figure 2).

### Justification for CTPA

We assessed whether the use of CTPA is justified in those with low or intermediate pre-test probability by the finding of an alternative diagnosis that might explain the patient’s symptoms. The frequency of alternate diagnoses found on CTPA was as follows: ED: 10%, Medicine: 5%, Surgery: 3%; p = 0.08. When we examine the data more closely, only 3.2% of potentially avoidable CTPA (low or intermediate pre-test probability and negative D-dimer) had an alternative diagnosis that was neither previously known nor evident on a chest radiograph performed prior to the CTPA.

### Findings on prior chest X-ray

13% of the patients did not have a prior chest X-ray defined as within 48 hours of CTPA. Among those who did undergo a chest X-ray, the findings were as follows: 40% normal, 14% pleural effusion, 11% pulmonary edema, 6% atelectasis, 6% infiltrate, and 10% other.

### Patients undergoing CTPA

In contrast to 2000 and 2005, those undergoing CTPA in 2008 were significantly younger and less acutely ill as evidenced by their higher oxygen saturation and lower respiratory rate (Table 1). Moreover, patients in 2008 were much less likely to have any risk factors for thrombosis or a prior history of venous thromboembolism. There was an increase in the use of CTPA in patients with chest pain, and for “other” indications (Table 1). The increase in number of scans ordered was accompanied by a significant drop in the diagnosis of PE from 22.6% in 2000 to 13% in 2010. We also noted a change in the size and location of pulmonary emboli detected over time: in 2008, a greater proportion of PE was found distal to the lobar arteries in the segmental or sub segmental branches (Table 2). Since younger patients and women may be more at risk from the potential carcinogenic effects of ionizing radiation [16], we also examined age and gender in our sample. The number of CTPA performed on women under the age of 40 years increased significantly over the study period (Table 1). While 11.8% of all CTPAs performed were on women less than 40 years of age, the presence of a PE in this cohort was lower than in all CTPAs reviewed (9.1% versus 17.7%).

**Table 1:** Demographics, signs and symptoms, risk factors and prior medical history by year for 850 patients undergoing CT angiography

**Characteristics**	**2000**	**2005**	**2008**	**Total**	**p**
***n***	74	383	393	850	
***Demographics***					
Age (years) (mean ± SD)	66.8 ± 16.8	62.9 ± 17	60 ± 19.3	61.9 ± 18.2	**0.004**
Female (%)	59.5	65	67.9	65.9	0.33
Females < 40 years (%)	6.8	7.8	16.5	11.8	**0.004**
Body mass index (kg/m^2^) (mean ± SD)	27.3 ± 5.5	27.4 ± 7.2	27.8 ± 6.2	27.6 ± 6.7	0.760
***Signs and symptoms***					
Shortness of breath (%)	79.7	63.7	62.3	64.5	**0.015**
Chest pain (%)	27	38.9	50.1	43.1	**<0.001**
Palpitations (%)	5.5	1.3	3.3	2.6	0.06
Syncope (%)	2.7	1	3.8	2.5	**0.04**
Hypoxia (%)	13.7	6.5	10.2	8.8	0.06
Cough (%)	9.5	9.9	12	10.8	0.60
Hemoptysis (%)	1.4	0.3	3.6	1.9	**0.003**
Lower extremity pain (%)	4.1	0	5.9	3.1	**<0.001**
Leg edema/tenderness (%)	1.4	0	8.9	4.2	**<0.001**
Other (%)	14.9	10.2	27.2	18.5	**<0.001**
Systolic blood pressure (mmHg)	128 ± 27	131 ± 23	129 ± 22	130 ± 23	0.389
Diastolic blood pressure (mmHg)	73 ± 13	75 ± 15	73 ± 14	74 ± 14	0.19
Respiratory rate (/min)	23.8 ± 7.5	20.7 ± 6	20 ± 5.7	20.6 ± 6	**<0.001**
Oxygen saturation (%)	92.7 ± 6.8	94.1 ± 6	95.3 ± 9.6	94.5 ± 8	**0.01**
Requiring supplemental O2 (%)	40.5	22.1	19	22.3	**<0.001**
***Risk factors for thrombosis (%)***					
Immobility	31.1	24.8	15.8	21.2	**0.001**
Active malignancy	31.1	29.8	22.1	26.4	**0.03**
Hospitalized in the prior 4 weeks	38.4	34.7	28.3	32.1	0.08
Surgery in the prior 4 weeks	25.7	17	16.3	17.4	0.14
Prior PE or DVT	17.6	9.4	12.5	11.5	**0.01**
Pregnant	1.4	1.6	5.1	3.2	**0.01**
Known hypercoagulable disorder	0	1.6	1	1.2	0.48
On estrogen therapy	6.8	1.8	3.6	3.1	0.06
***Prior medical history (%)***					
Any lung disease	29.7	26.4	24.4	25.8	0.58
Hypertension	48.7	16.5	51.2	35.3	**<0.001**
Coronary artery disease	27	18.5	23.2	21.4	0.14
Congestive heart failure	18.9	6	6.6	7.4	**<0.001**
Other cardiac disease	25.8	6.8	13.5	11.5	0.26
Dialysis	0	1.3	2.3	1.7	0.28
Transferred from another institution	8.2	11.5	8.4	9.8	0.31

**Table 2:** CTPA positivity rate and location of pulmonary emboli

**CTPA Results**	**2000**	**2005**	**2008**	**Total**	**p**
***n***	74	383	393	850	
***Embolus present (%)***	25.7	19.1	14.8	17.7	**0.048**
***Location of embolus for positive studies (%)***					
Central PE	89.6	46.6	51.8	53.9	**0.003**
Peripheral PE	10.5	53.4	48.3	46	

Central PE was defined as PE in the pulmonary trunk, right or left main pulmonary arteries or lobar arteries while PE in segmental or sub-segmental branches were considered peripheral.

There was no correlation between pre-test probability by RGS and location or size of PE.

### Pre-test probability

Across all years, physicians ordering CTPA rarely documented the pre-test probability of PE in the patients’ medical records (Table 3). Our calculation of pre-test probability based on the RGS showed that more patients with low pre-test probability of PE underwent CTPA in later years (Table 3). Overall, PE was present on CTPA in 9.3% of patients with a low pre-test probability, 20.9% patients with an intermediate pre-test probability and 29.6% with a high pre-test probability of PE. Slightly over one fifth (22.2%) of patients with a high pre-test probability of PE received any form of anticoagulation prior to undergoing CTPA.

**Table 3:** Assessment of clinical pre-test probability and RGS

	**2000**	**2005**	**2008**	**Total**	**p**
***n***	74	383	393	850	
***Pre-test probability of PE documented (%)***	4.1	1.6	3.1	2.4	0.48
***Calculated Revised Geneva Score (mean ± SD)***	5.7 ± 2.9	5.3 ± 2.5	5.4 ± 3.0	5.4 ± 2.8	0.42
Low probability (%)	25.7	24.5	28.8	26.6	**0.01**
Intermediate probability (%)	70.3	74.4	66.2	70.2	
High probability (%)	4.1	1	5.1	3.2	

### Use of D-dimer

D-dimer was assessed in an increasing number of patients with low or intermediate pre-test probability of PE. In 2000, no patient with a low or intermediate RGS underwent a D-dimer test. In 2005 this number was 21.3% and in 2008 was 31.9%. Notably, our data show that when the D-dimer was negative in such patients and PE was not diagnosed on CTPA (Table 4). Among those with high pre-test probability, the D-dimer was always positive when performed.

**Table 4:** Use of D-dimer in patients undergoing CTPA

**Pre-test probability**	**n**	**D-dimer not performed, % (CTPA positive %)**	**D-dimer performed**	
			**Negative D-dimer, % (CTPA positive, %)**	**Positive D-dimer, % (CTPA positive, %)**
***Low***				
2000	19	100.0 (100)	-	-
2005	94	78.7 (6.8)	4.3 (0)	17 (0)
2008	113	68.1 (16.9)	7 (0)	24.8 (7.1)
***Intermediate***				
2000	52	98.1 (33.3)	1.9 (0)	-
2005	285	81.0 (23.8)	1.8 (0)	17.2 (24.5)
2008	260	79.2 (15.5)	4.6 (0)	16.2 (21.4)
***High***				
2000	3	100.0 (66.7)	-	-
2005	4	75.0 (33.3)	-	25 (100)
2008	20	85.0 (23.5)	-	15 (0)

### V/Q scanning

**Figure 3:**
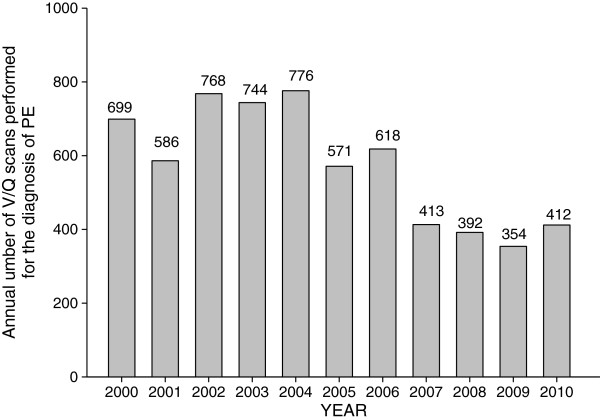
Number of ventilation-perfusion scans (V/Q) performed for the diagnosis of PE from 2000 to 2012.

Since the increase in CTPA may be mirrored by a corresponding decrease in V/Q scans, we were interested as to whether the number of V/Q performed for the diagnosis of PE decreased accordingly (Figure 3). The installation and availability of another CT scanner in 2005 resulted in 19% drop in V/Q scans compared to 2000. By 2008, the number of V/Q scans being performed for the diagnosis of PE dropped by 31% compared to 2005 (Table 5), and by 44% compared to 2000. Table 5 also illustrates the changes in ED visits and hospital admissions for the index years 2000, 2005 and 2008.

**Table 5:** Trend in number of CTPAs and V/Q scans performed, as well as ED visits and hospital admissions for the years 2000, 2005 and 2008

	**2000**	**2005**	**2008**	**% change 2000 - 2005**	**% change 2005 - 2008**
**CTPA**	84	1114	1883	1226	69
**V/Q scan**	699	571	392	−18	−31
**ED visits**	41,464	41,004	48,813	−1	19
**Hospital admissions**	42,483	41,398	45,770	−3	10

## Discussion

From the year 2000 to 2010, the total number of CTPA performed at our center increased exponentially although the percentage of scans positive for PE declined. This increase in CTPA was not accompanied (or justified) by finding an alternative diagnoses on CTPA that explains the patients’ symptoms. Indeed, our data indicate that CTPA rarely reveals a previously unknown or new finding especially in those with low and intermediate risk for PE. CTPA provided an alternate diagnosis, defined as one that was not previously known or evident on chest radiograph in only 7.6% of all non-PE CTPA. Additionally, this number would drop to 3.2% had the published guidelines for investigation of suspected PE been followed, that is in those with low or intermediate pre-test probability and a negative D dimer. We also find that CTPAs were being performed in younger patients even in the absence of symptoms and identifiable PE risk factors further negating the argument of discovering an alternative pathological diagnosis. Finally, the increase in the number of CTPA corresponds neither to the decrease in the number of V/Q scans nor to the increase in the number of ED visits or hospital admissions.

The utility of CTPA to provide an alternative diagnosis is often cited as justification for obtaining this test even when PE is not found [9-15]. In prior studies, the most frequently cited alternative diagnosis on CTPA is an infiltrate or consolidation suggestive of pneumonia. However, it is not recorded whether a history and physical examination had already suggested pneumonia; neither was it noted whether a chest radiogram was performed prior to the CTPA. While it is difficult to compare studies as purpose, setting and design differ, one retrospective study reported a third of CTPAs revealed an alternative explanation of the patient’s presenting symptoms but such findings were already known in over half of patients from the admission chest radiogram [9,17]. In our center, 40% of the chest X-rays performed prior to the CTPA were interpreted as normal, while in 13% of patients a chest film was not obtained within 48 hours of ordering the CTPA. This indicates that in just fewer than half the patients (47%) a pathological diagnosis was present on the chest film. Clearly the presence of a radiological process on chest X-ray does not rule out the concomitant presence of PE, and we cannot determine what the ordering physician was considering. However the combination of diminishing yield of the CTPA from 2000 to 2010, the younger and healthier patients on whom CTPA is being performed, the underutilization of pre-tests probability and D-dimer, the presence of known lung pathology on a chest film, the drop in V/Q scans not matched by the increase in the number of CTPAs, all attest to the unjustified overuse of CTPA.

Accepted recommendations for the diagnosis of PE continue to emphasize pre-test probability to guide the choice of testing, and the positive predictive value of CTPA remains dependent on the pre-test probability of PE [3-5]. We used documentation of pre-test probability in patients’ medical records as a surrogate for assignment of pre-test probability by any means, subjective or objective. It is possible that assessment of pre-test probability was performed by physicians prior to CTPA, but not specifically recorded. However, if this were the case, such assessment of pre-test probability occurred in a rather small minority of patients for the following two reasons. First, D-dimer was only measured in 20% of all patients with a low or intermediate pre-test probability of PE. Second, only 1 in 5 patients in the high pre-test probability category received anticoagulation while awaiting confirmation of PE with a CTPA. Our study was designed to investigate the rates and indications for the use of CTPA in clinical practice and therefore only includes patients undergoing CTPA. It is possible that patients presented with low risk for PE, were appropriately screened and did not undergo CTPA. We believe that such number is small as our data reveal that younger patients with fewer risk factors for PE were undergoing CTPA.

We assigned pre-test probability retrospectively using the RGS that tends to classify the great majority of patients in the low or intermediate categories and this could limit the interpretability of our findings. However, the limitation of the RGS notwithstanding, it is suited for retrospective calculation and variables included in the RGS can be accurately assessed by systematic chart review (25).

The British Thoracic Society’s guidelines advise that should a PE be suspected, the patient should be fully evaluated by an experienced middle-grade doctor (the equivalent of a resident in their third year of training) so that alternative diagnoses are considered and clinical probability for PE is documented. Such practice should then yield a 25% incidence of PE when CTPA is performed [7]. Our data show that CTPA yields a positive diagnosis in only 11% to 15% of all CTPAs. Though subjective assessment by experienced physicians has been shown to be non-inferior to objective assignment of pre-test probability, unfortunately, the present trend in ordering CTPA could make it unlikely for such expertise in the clinical diagnosis of PE to develop. Not only are recommendations not being followed in routine clinical practice, but a greater proportion of CTPA are being performed in patients with lower pre-test probability and on younger and seemingly healthier patients. As guidelines outline how to best utilize CTPAs [3-5,14] and articles lament the over use of CTPAs [8,17-21], Glaser and colleagues demonstrated that a simpler reporting strategy for V/Q scans (PE present, PE absent and non-diagnostic) can be safely implemented, facilitates clearer communication with referring clinicians and may reduce the number of CTPAs ordered [22].

It is possible that the results of this study are isolated to our medical center; however, this is highly suspect. There is little reason to indicate that practice at our center would differ from national practice, especially upon reviewing the literature. Other studies, albeit smaller and covering a shorter time span, are consistent with a dramatic increase in CTPA use [6,7]. Yin and colleagues [23] also found that D-dimer assay was not being used appropriately in a one year evaluation of CTPAs performed in their institution. The increase in CT utilization by our ED is by no means unique; indeed the overall use of CT scan in the ED had risen by 14% a year since 1995 and by 330% from 1996 to 2007 [18,24].

## Conclusion

In conclusion, our data indicate that search for an alternate radiologic diagnosis does not justify what appears to be an indiscriminate use of CTPA. CTPA is being increasingly used as the first and only diagnostic test for suspected PE and the frequency of positive CTPAs has declined significantly over time, with younger and healthier patients being tested.

## Competing interest

The authors declare that they have no conflict of interests relating to this work.

## Authors’ contributions

SC participated in study design, data collection and entry and drafted the manuscript. PS participated in data collection and entry and contributed to study design. DC performed the statistical analysis and contributed to the manuscript. NG participated in data collection and entry. RC conceived of the study, and participated in its design and coordination and contributed to the manuscript. All authors read and approved the final manuscript.

## Pre-publication history

The pre-publication history for this paper can be accessed here:

http://www.biomedcentral.com/1471-2466/13/9/prepub
